# Age-dependent postoperative cognitive impairment and Alzheimer-related neuropathology in mice

**DOI:** 10.1038/srep03766

**Published:** 2014-01-20

**Authors:** Zhipeng Xu, Yuanlin Dong, Hui Wang, Deborah J. Culley, Edward R. Marcantonio, Gregory Crosby, Rudolph E. Tanzi, Yiying Zhang, Zhongcong Xie

**Affiliations:** 1 Geriatric Anesthesia Research Unit, Department of Anesthesia, Critical Care and Pain Medicine, Massachusetts General Hospital and Harvard Medical School, Charlestown, MA 02129-2060; 2 Department of Anesthesia, Beijing Chaoyang Hospital, Capital Medical University, Beijing, P. R. China 100020; 3 Department of Anesthesia, Brigham & Women's Hospital and Harvard Medical School, Boston, MA 02115; 4 Divisions of General Medicine and Primary Care and Gerontology, Department of Medicine, Beth Israel Deaconess Medical Center and Harvard Medical School, Boston, MA 02115; 5 Genetics and Aging Research Unit, MassGeneral Institute for Neurodegenerative Disease, Department of Neurology, Massachusetts General Hospital and Harvard Medical School, Charlestown, MA 02129-2060; 6 These authors contributed equally to this work.

## Abstract

Post-operative cognitive dysfunction (POCD) is associated with increased cost of care, morbidity, and mortality. However, its pathogenesis remains largely to be determined. Specifically, it is unknown why elderly patients are more likely to develop POCD and whether POCD is dependent on general anesthesia. We therefore set out to investigate the effects of peripheral surgery on the cognition and Alzheimer-related neuropathology in mice with different ages. Abdominal surgery under local anesthesia was established in the mice. The surgery induced post-operative elevation in brain β-amyloid (Aβ) levels and cognitive impairment in the 18 month-old wild-type and 9 month-old Alzheimer's disease transgenic mice, but not the 9 month-old wild-type mice. The Aβ accumulation likely resulted from elevation of beta-site amyloid precursor protein cleaving enzyme and phosphorylated eukaryotic translation initiation factor 2α. γ-Secretase inhibitor compound E ameliorated the surgery-induced brain Aβ accumulation and cognitive impairment in the 18 month-old mice. These data suggested that the peripheral surgery was able to induce cognitive impairment independent of general anesthesia, and that the combination of peripheral surgery with aging- or Alzheimer gene mutation-associated Aβ accumulation was needed for the POCD to occur. These findings would likely promote more research to investigate the pathogenesis of POCD.

Each year, about one to two million Americans over 65 years of age suffer from post-operative cognitive dysfunction (POCD), which is one of the most common post-operative complications in senior patients^1^ and is associated with increased cost, morbidity, and mortality^2^,^3^,^4^. However, the causes and pathogenesis of POCD remain largely to be determined.

Previous studies have assessed the effects of general anesthesia or surgery plus general anesthesia on cognitive impairment in rodents^5^,^6^,^7^,^8^. But there is increasing clinical evidence which suggests that surgery in the absence of general anesthesia may also induce POCD in humans^9^. Therefore, it is important to determine whether POCD in humans and cognitive impairment in animals are dependent on the presence of general anesthesia.

It has been reported that surgery may cause neuroinflammation, including elevation of the levels of pro-inflammatory cytokine e.g., TNF-α^7^, and activation of microglia^8^, leading to POCD [reviewed in^10^]. However, almost all surgical patients develop a certain degree of inflammation and some surgical patients may develop neuroinflammation, the majority of surgical patients do not develop POCD. The reason behind this observable fact is largely unknown. Excessive accumulation of β-amyloid (Aβ) has been reported as a part of the neuropathogenesis of Alzheimer's disease (AD) and cognitive impairment (reviewed in^11^). We have therefore postulated a multifactorial model of POCD pathogenesis that peripheral surgery (precipitating factors) plus Aβ accumulation from aging [e.g., 18 month-old wild-type (WT) mice] or AD gene mutation [e.g., 9 month-old AD transgenic (Tg) mice] (predisposing factors) were needed to cause the cognitive impairment in mice.

Therefore, we established a pre-clinical model of peripheral surgery in the abdomen under local anesthesia to determine the effects of peripheral surgery without the influence of general anesthesia on Aβ accumulation and cognitive impairment in 9 and 18 month-old WT mice, and 9 month-old AD Tg mice. The studies aimed to: (1) establish a pre-clinical model of POCD without the presence of general anesthetics to assess whether POCD was independent of general anesthetics; and (2) elucidate the pathogenesis of POCD by investigating whether the peripheral surgery could induce an age-dependent Aβ accumulation and cognitive impairment. The AD Tg mice [B6.Cg-Tg(APPswe, PSEN1dE9)85Dbo/J] have the same genetic background as the WT mice (C57BL/6J) and elevated Aβ levels, owing to mutations of *APP* and *PSEN1*, the AD genes^12^,^13^].

Aβ is generated from its large precursor protein amyloid precursor protein (APP) by sequential proteolytic cleavage through two proteases, beta-site APP cleaving enzyme (BACE1) and γ-secretase (reviewed in^11^). Cellular stress may enhance phosphorylation of the eukaryotic translation initiation factor (eIF) 2α, leading to increases in levels of BACE1 and consequently Aβ accumulation^14^. γ-Secretase inhibitor compound E can reduce Aβ generation^15^. We therefore determined the effects of the peripheral surgery on the brain level of BACE1 and phosphorylated eIF2α, and assessed whether compound E could attenuate the peripheral surgery-induced cognitive impairment and brain Aβ accumulation in the 18 month-old WT mice.

## Results

**Figure 1:**
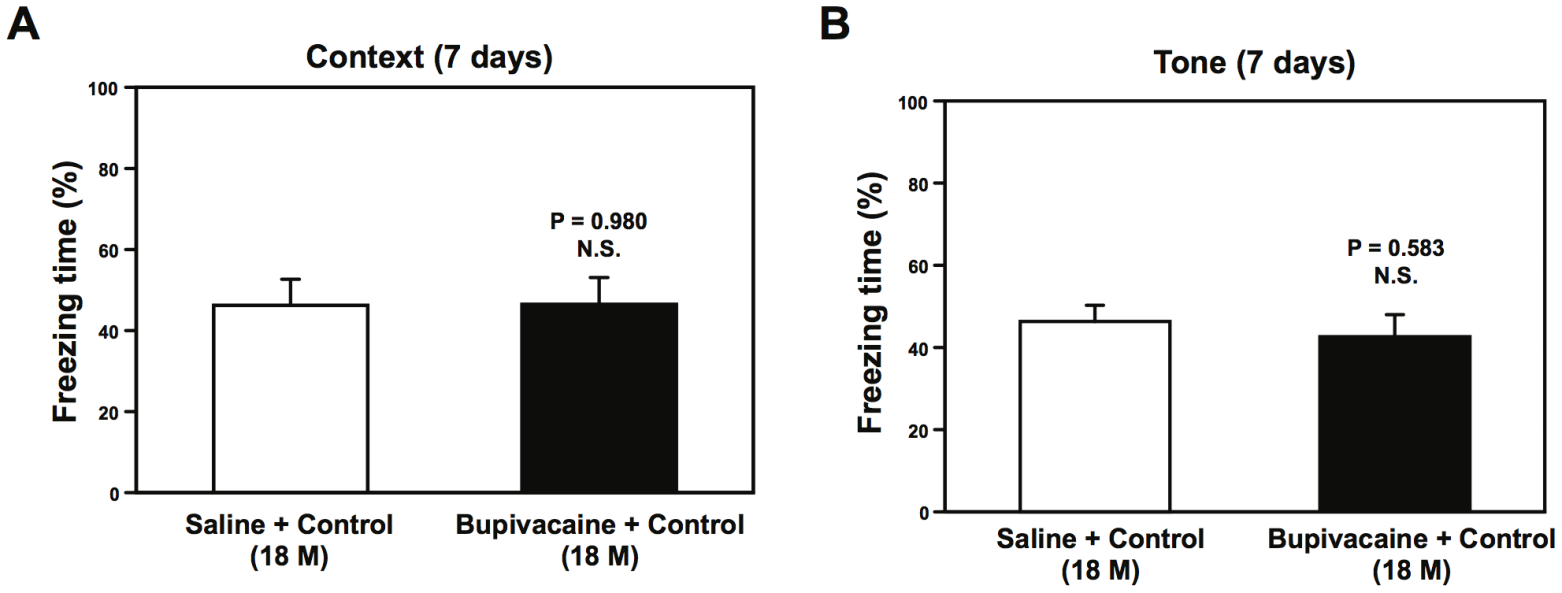
Local anesthesia does not induce cognitive impairment in the mice. Local anesthesia with bupivacaine does not induce cognitive impairment in context test (A) and tone test (B) of FCS in 18 month-old mice 7 days post-surgery. N = 10. FCS, Fear Conditioning System.

The mice that received the peripheral surgery did not show significant changes in behavior (e.g., eating and drinking), intraoperative blood pressure, blood gas, blood glucose and epinephrine levels, pain threshold, and post-operative locomotor activity as compared to the control mice (Table 1). Mice had the surgical procedure under bupivacaine local anesthesia. The local anesthesia alone did not induce cognitive impairment in the aged mice (18 month-old mice) at 7 days after the abdominal surgery (Figure 1).

### The peripheral surgery in the absence of general anesthesia induced cognitive impairment in aged WT mice

**Figure 2:**
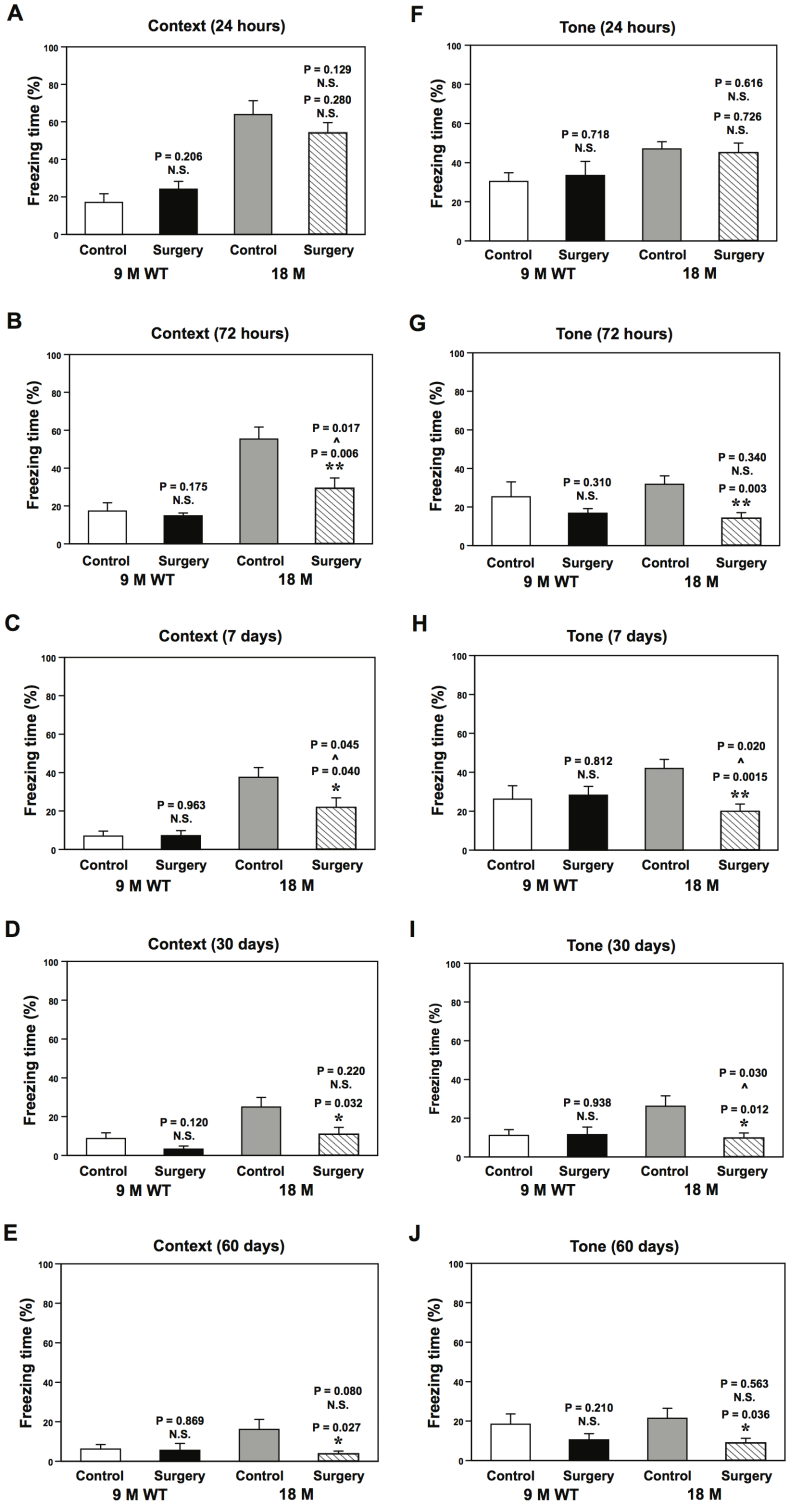
Peripheral surgery impairs associative cognitive function in 18, but not 9, month-old WT mice. Peripheral surgery decreases freezing time in the context test of the FCS (* P < 0.05 or ** P < 0.01) at 72 hours (B), 7 (C), 30 (D) and 60 (E) days, but not 24 hours (A), post-surgery in 18 month-old WT mice, but not in 9 month-old WT mice. Two-way ANOVA shows that age potentiates the peripheral surgery-induced cognitive impairment (context test, ^∧^ P < 0.05) at 72 hours (B) and 7 days (C) post-surgery. Peripheral surgery decreases freezing time in the tone test of the FCS (* P < 0.05 or ** P < 0.01) at 72 hours (G), 7 (H), 30 (I) and 60 (J) days, but not 24 hours (F), post-surgery in 18 month-old WT mice, but not in 9 month-old WT mice. Two-way ANOVA shows that age potentiates the peripheral surgery-induced cognitive impairment (tone test, ^∧^ P < 0.05) at 7 (H) and 30 (I) days post-surgery. N = 10. FCS, Fear Conditioning System; wild-type, WT; analysis of variance, ANOVA.

Fear Conditioning System (FCS) is among the most commonly used behavioral tests to detect cognitive impairment induced by anesthesia^5^,^6^ and anesthesia plus surgery^7^,^8^. We therefore first assessed and compared the effects of peripheral surgery without general anesthetics (under the local anesthesia) on cognitive function in adult (9 month-old) and aged (18 month-old) WT mice using the FCS. We found that the peripheral surgery in the absence of general anesthesia impaired cognitive function as evidenced by reductions in freezing time in both context and tone tests of the FCS at 72 hours, 7, 30, and 60 days, but not 24 hours, post-surgery in 18, but not 9, month-old WT mice (Figure 2). Two-way ANOVA showed that age potentiated the peripheral surgery-induced cognitive impairment (Figure 2). The relatively short freezing time that 9 month-old WT mice exhibited in the context test of the FCS could be due to a hyperactive response of the mice while in the FCS chamber.

**Figure 3:**
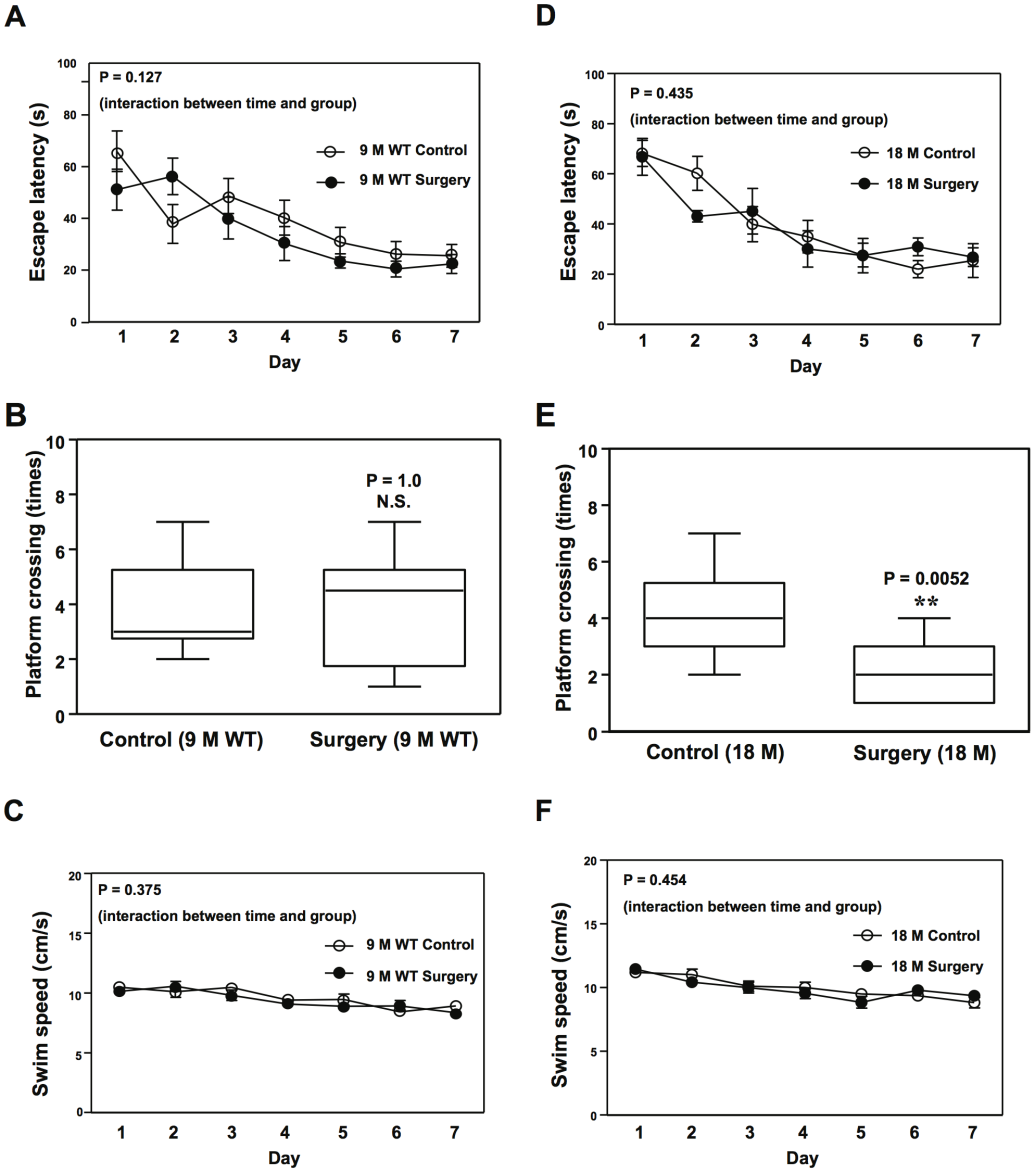
Peripheral surgery impairs spatial cognition in 18 month-old WT mice. The peripheral surgery, in the absence of general anesthetics, induces neither an increase in escape latency nor a decrease in platform crossing times in 9 month-old WT mice (A and B) in the MWM test. In the 18 month-old WT mice, however, the peripheral surgery decreases platform crossing times: 4, 3–5.25 (median, interquartile range) versus 2, 3–1 (median, interquartile range), ** P = 0.0052 (E) in the MWM test. The peripheral surgery does not increase escape latency (D) in the 18 month-old WT mice in the MWM studies. There is no significant difference in swim speed between the control and surgery conditions in 9 month-old WT (C) or 18 month-old WT mice (F). Morris Water Maze, MWM; wild-type, WT. Values are expressed as mean ± SEM. N = 10.

Moreover, the peripheral surgery reduced the number of times that mice crossed the platform in the probe test of the Morris Water Maze in 18 (Figure 3E), but not 9 (Figure 3B), month-old WT mice. The peripheral surgery did not significantly alter the escape latency (Figure 3A and 3D) and swim speed (Figure 3C and 3F) of the mice in the MWM test.

Collectively, these data indicated that the peripheral surgery without the influence of general anesthetics induced associative^7^,^8^ and spatial^16^ impairment of cognitive function in aged mice.

### The peripheral surgery increased hippocampus Aβ levels in aged WT mice

It has been reported that the neuroinflammation following surgery may be associated with cognitive impairment in animals^7^,^8^ and in patients^17^. However, even though all patients may have a peripheral surgery-induced increase in pro-inflammatory cytokines in the blood (which could cause neuroinflammation^18^), not all patients develop POCD. Thus, it is plausible that patients who develop POCD have other changes in the brain that facilitate cognitive impairment. We have hypothesized that one of these changes is an elevated level of brain Aβ, and therefore assessed the effects of the peripheral surgery on the Aβ levels in the hippocampus of the mice.

**Figure 4:**
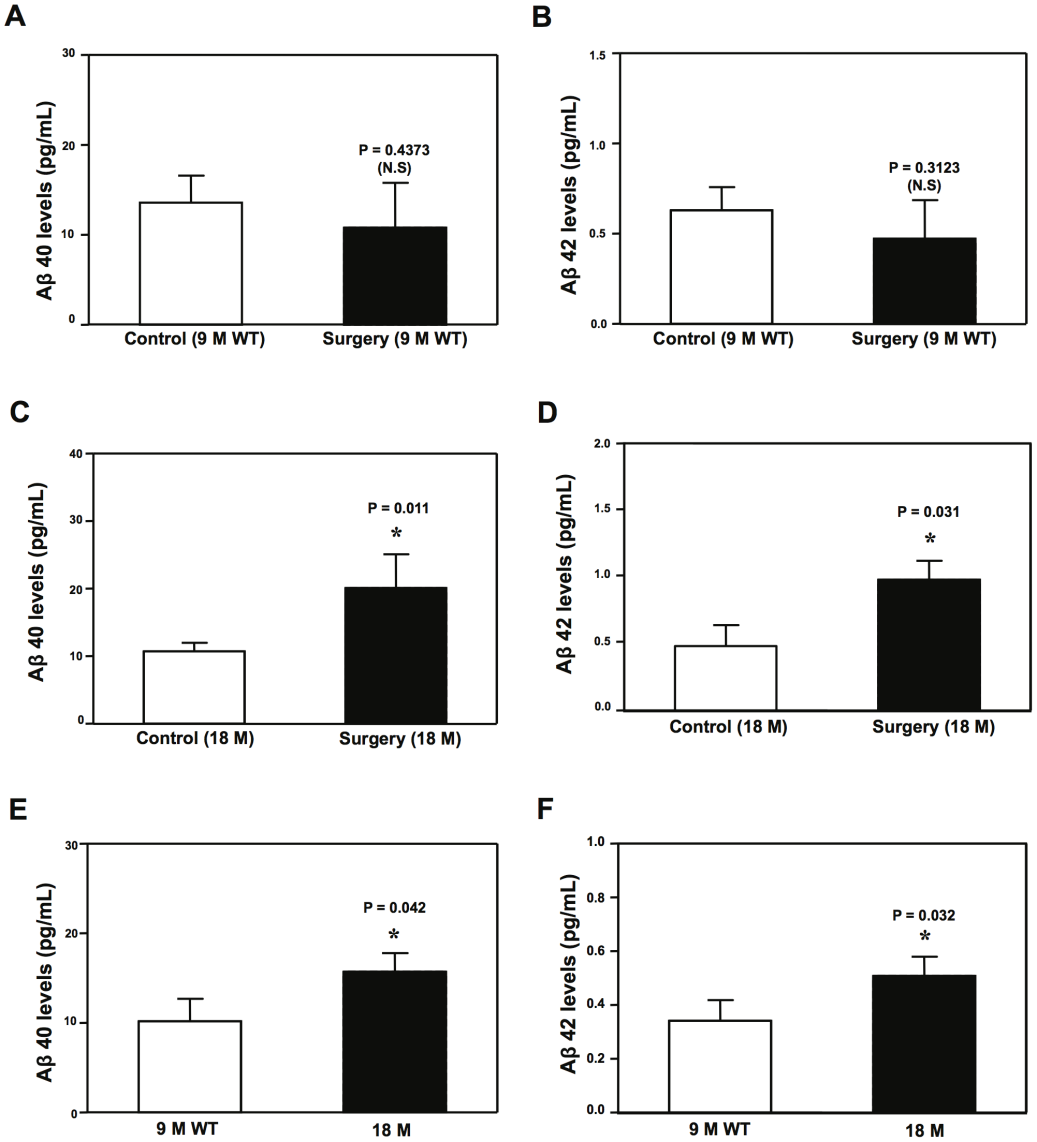
Peripheral surgery increases Aβ levels in the hippocampus of 18 month-old WT mice. ELISA shows that the peripheral surgery does not increase the levels of Aβ40 (A) and Aβ42 (B) in the hippocampus of 9 month-old WT mice. Peripheral surgery significantly increases the levels of Aβ40 (C) and Aβ42 (D) in the hippocampus of 18 month-old WT mice. ELISA shows that there are higher baseline levels of Aβ40 (E) and Aβ42 (F) in the hippocampus of 18 month-old WT mice than those of 9 month-old WT mice. β-Amyloid protein, Aβ; wild-type, WT. N = 6–8.

Enzyme-linked immunosorbent assay (ELISA) of Aβ showed that the peripheral surgery (black bar) did not significantly increase the levels of Aβ40 (Figure 4A) and Aβ42 (Figure 4B) in the hippocampus of 9 month-old WT mice as compared to the control condition (white bar). In the 18 month-old WT mice, however, the peripheral surgery significantly increased Aβ levels in the hippocampus of the mice 12 hours post-surgery (Figure 4C and Figure 4D). Finally, the ELISA showed that the baseline levels of Aβ in the hippocampus of the 18 month-old WT mice were higher than those in the 9 month-old WT mice (Figure 4E and 4F). These data suggested that the peripheral surgery increased both Aβ40 and Aβ42 levels in the hippocampus of aged WT mice, but not in the adult WT mice. These results were consistent with the finding from the previous studies that aging is associated with elevated brain Aβ levels^19^.

### The peripheral surgery induced cognitive impairment and enhanced hippocampus Aβ levels in adult AD Tg mice

**Figure 5:**
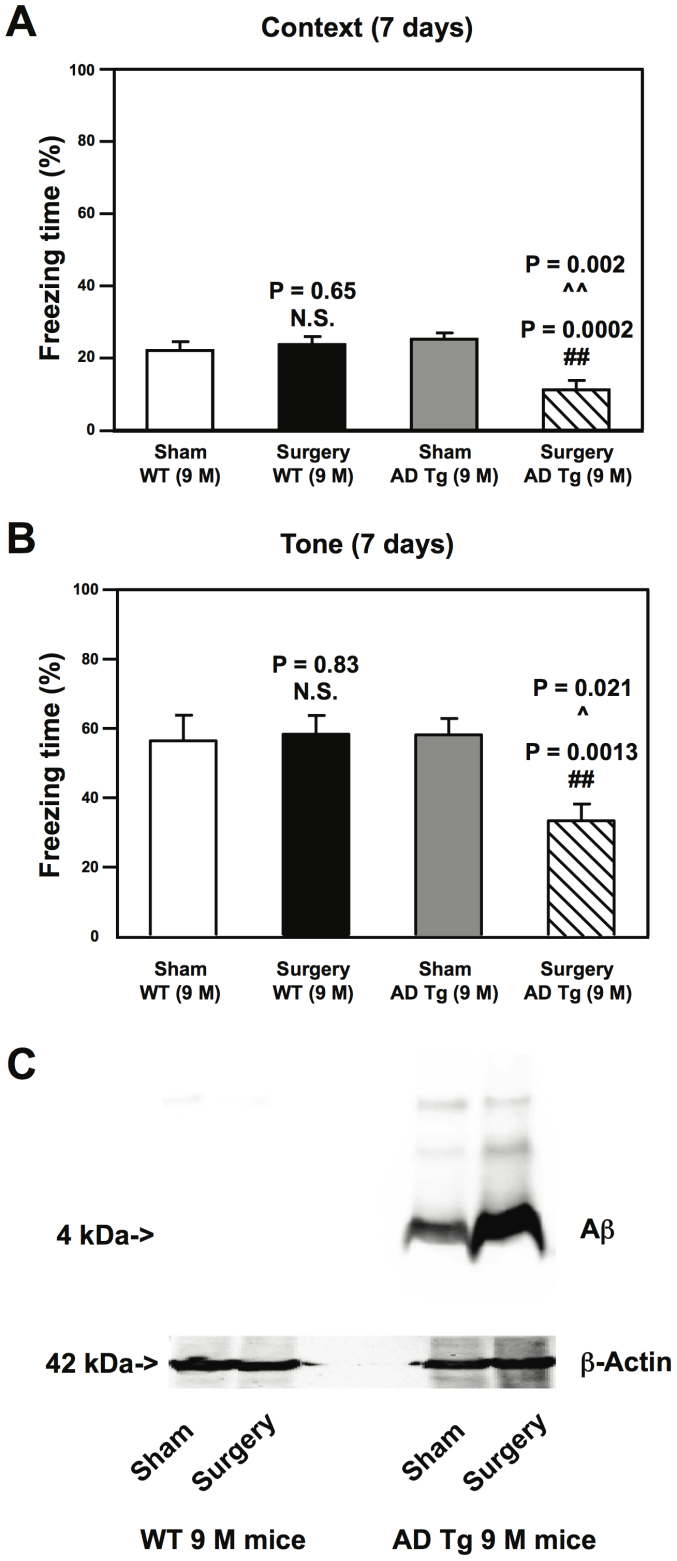
Peripheral surgery impairs cognitive function and increases hippocampus Aβ levels in 9 month-old AD Tg mice but not in 9 month-old WT mice. Peripheral surgery, in the absence of general anesthetics, decreases freezing time in the context test (A) and tone test (B) of the FCS at 7 days post-surgery in 9 month-old AD Tg mice but not in 9 month-old WT mice. Two-way ANOVA shows that AD gene mutations (*APP* and *PSEN1*) potentiate the peripheral surgery-induced cognitive impairment at 7 days post-surgery: context test, ^∧∧^ P = 0.002; tone test: ^∧^ P = 0.021. N = 10. (C). The baseline Aβ levels in the hippocampus of the 9 month-old AD Tg mice are higher than those in 9 month-old WT mice, and the peripheral surgery increases the hippocampus Aβ levels in the 9 month-old AD Tg mice but not in the 9 month-old WT mice. Alzheimer's disease, AD; β-amyloid protein, Aβ; transgenic, Tg; wild-type, WT; analysis of variance, ANOVA; amyloid protein precursor, APP; presenilin 1, PSEN1.N = 10 (behavioral tests), N = 6 (biochemistry studies, but only one sample was used to represent the findings). Full-length blots/gels are presented in Supplementary Figure 1.

Next, we employed AD Tg mice to further test the hypothesis that the peripheral surgery exclusively induced cognitive impairment and enhanced brain Aβ levels in the mice with elevated baseline brain Aβ levels. We found that the peripheral surgery induced cognitive impairment (Figure 5A and 5B) and Aβ accumulation (Figure 5C) in the 9 month-old AD Tg mice [B6.Cg-Tg(APPswe, PSEN1dE9) 85Dbo/J], but not in the 9 month-old WT mice, 7 days and 12 hours post-surgery, respectively. The AD Tg mice [B6.Cg-Tg (APPswe, PSEN1dE9)85Dbo/J] have elevated Aβ levels^12^,^13^]. Taken together, these data further suggested that the peripheral surgery only enhanced brain Aβ accumulation and induced cognitive impairment in the mice with already elevated baseline brain Aβ levels. Note that the freezing time of mice in the tone test of FCS in this experiment when the mice were tested once 7 days after the surgery (Figure 5A) was higher than that in the experiment when mice were tested repeatedly in FCS at 24 hours, 72 hours, 7 days, 30 days and 60 days after the surgery (Figure 2). The exact reason of such difference is unknown at the present time. We postulate that mice may have reductions in the freezing time of FCS tone test when they are tested repeatedly in FCS after surgery. The future studies to test this hypothesis are warranted.

### The peripheral surgery increased levels of BACE1 and P-eIF2α in hippocampus of aged WT mice

**Figure 6:**
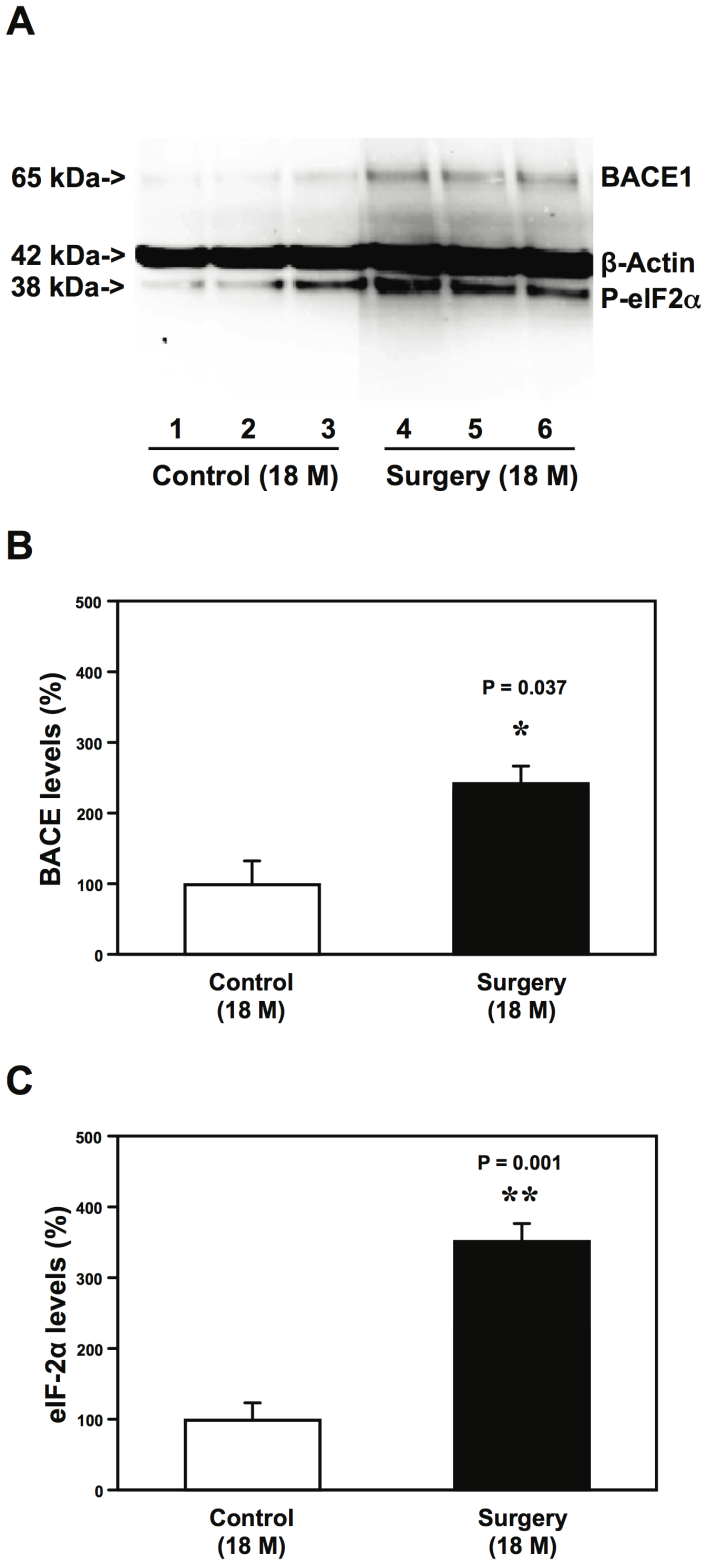
Peripheral surgery increases the levels of BACE1 and P-eIF2α in mouse hippocampus. (A). Peripheral surgery (bands 4 to 6) increases BACE1 and P-eIF2α levels in the hippocampus of 18 month-old mice at 12 hours post-surgery as compared to control condition (bands 1 to 3). Quantification of the Western blot shows that the peripheral surgery (black bar) increases BACE1 (B) and P-eIF2α (C) levels in the hippocampus of 18 month-old WT mice at 12 hours post-surgery as compared to the control condition (white bar). Beta-site amyloid precursor protein cleaving enzyme, BACE1; phosphorylated eukaryotic translation initiation factor 2α, P-eIF2α. Full-length blots/gels are presented in Supplementary Figure 2.

Cellular stress has been reported to enhance phosphorylation of the eukaryotic translation initiation factor (eIF) 2α, which then lead to increases in levels of BACE1 and consequently Aβ accumulation^14^. We therefore assessed the effects of the peripheral surgery without the influence of general anesthetics on the brain levels of BACE1 and phosphorylated eIF2α (P-eIF2α) in mice. Quantitative Western blot showed that the peripheral surgery increased the levels of BACE1 and P-eIF2α in the hippocampus of 18 month-old WT mice 12 hours post-surgery (Figure 6A, 6B and 6C). These data suggested that the peripheral surgery might induce Aβ generation by increasing the levels of P-eIF2α and BACE1.

### Compound E attenuated the peripheral surgery-induced brain Aβ accumulation and cognitive impairment in aged mice

**Figure 7:**
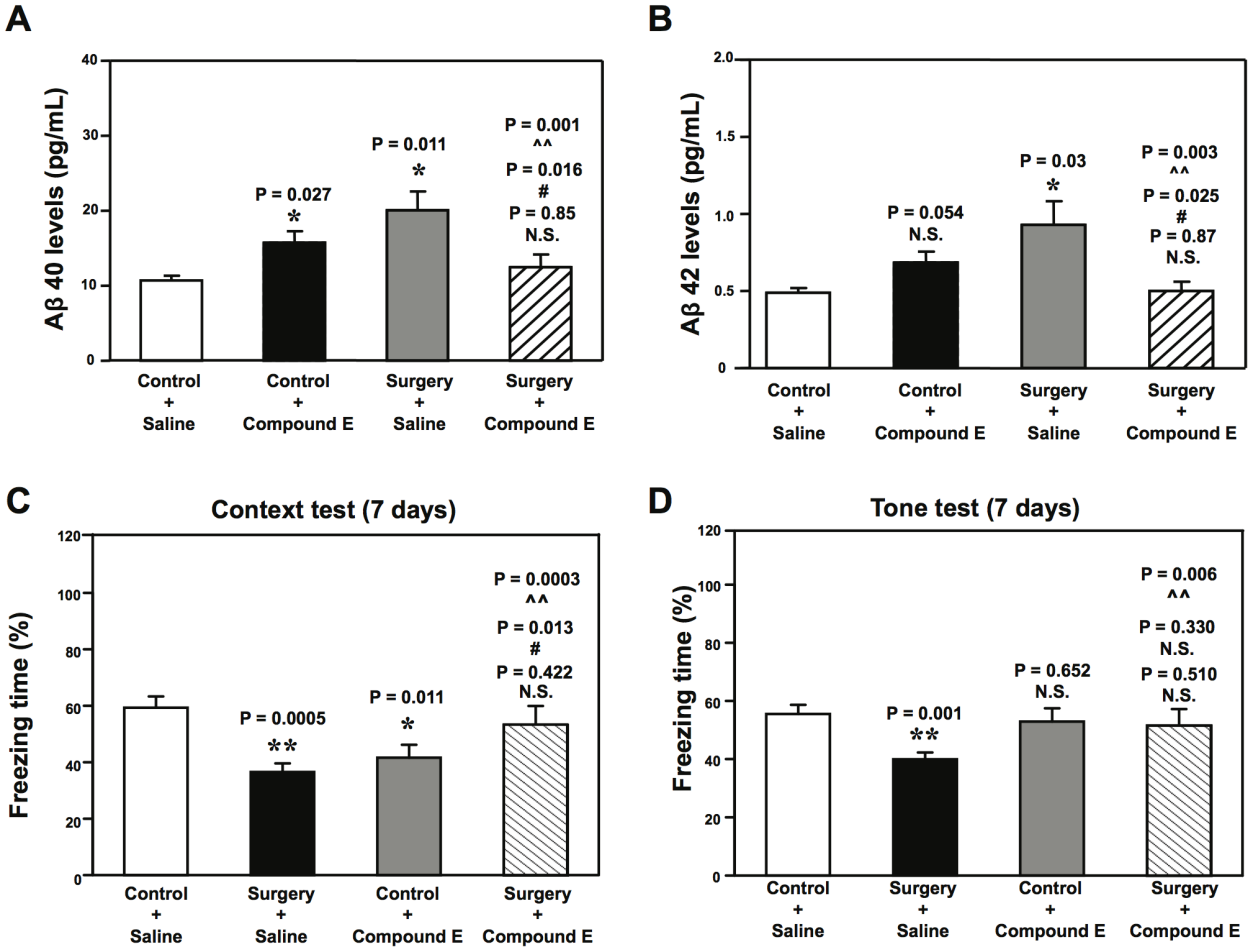
Compound E attenuates the peripheral surgery-induced increases in levels of Aβ in the hippocampus, and ameliorates the peripheral surgery-induced cognitive impairment in aged WT mice. Compound E (3 mg/kg/day for 7 days) mitigates the peripheral surgery-induced increases in levels of Aβ in the hippocampus of 18 month-old WT mice 12 hours post-surgery (* or **: the difference between control and peripheral surgery group; #: the difference between saline and compound E treatment; ^∧^ or ^∧∧^: the interaction between the group and the treatment) (A and B). Compound E ameliorates the peripheral surgery-induced cognitive impairment in 18 month-old WT mice at 7 days post-surgery (C and D) (* or **: the difference between control and peripheral surgery group; #: the difference between saline and compound E treatment; ^∧∧^: the interaction between the group and the treatment). β-Amyloid protein, Aβ; wild-type, WT. N = 6 (biochemistry studies) and N = 10 (behavioral studies).

Given that the peripheral surgery without the influence of general anesthetics increased Aβ accumulation and induced cognitive impairment in aged mice, next we assessed the cause-effect relationship by employing compound E, a γ-secretase inhibitor that decreases Aβ generation^20^. We found that compound E attenuated the peripheral surgery-induced increase in the levels of Aβ40 (Figure 7A) and Aβ42 (Figure 7B) in the hippocampus of 18 month-old mice 12 hours post-surgery. Finally, compound E ameliorated the peripheral surgery-induced cognitive impairment (Figure 7C and 7D) 7 days post-surgery. These results further suggested that the peripheral surgery likely induced cognitive impairment by enhancing brain Aβ accumulation in the aged mice.

## Discussion

Many studies aim to determine the role of general anesthesia alone (6,21,22; reviewed in^23^) or general anesthesia plus surgery^8^,^24^,^25^,^26^ in POCD pathogenesis. However, increasing evidence suggests that there is no significant difference in the incidence of POCD between surgery with general anesthesia and surgery without it (with epidural, spinal, or local anesthesia) (27,28; reviewed in^29^). We therefore established a pre-clinical model in mice and aimed to determine whether POCD can occur even in the absence of general anesthetics. We found that the peripheral surgery in the abdomen, in the absence of general anesthetics (under local anesthesia), still caused cognitive impairment in aged WT mice (Figure 2 and 3). These results suggested that POCD may not be dependent on the presence of general anesthetics. However, it is still possible that general anesthetics may potentiate the surgery-induced POCD in humans and cognitive impairment in animals. Further studies should include comparing the effects of anesthesia, surgery, and anesthesia plus surgery on cognitive function and the underlying mechanisms.

Moreover, we found that the peripheral surgery increased Aβ levels exclusively in the hippocampus of aged WT mice (Figure 4) or AD Tg mice (Figure 5), but not adult mice, which was paralleled with the cognitive impairment in the mice (Figure 2, 3 and 5). The hippocampus baseline Aβ levels in the aged WT mice were higher than adult WT mice (Figure 4), and the hippocampus baseline Aβ levels in AD Tg mice were higher than those in WT mice (Figure 5). Collectively, these data suggested a hypothesized multifactorial mode of POCD that the combination of peripheral surgery and Aβ accumulation from aging or AD gene mutation was needed to cause the cognitive impairment in mice. These data are consistent with the clinical observation that senior patients, who have higher brain Aβ levels^19^, are more vulnerable to develop POCD^30^. These findings were also consistent with the findings from a previous study that the partial hepatectomy in mice induced Aβ production^8^.

Cellular stress induced by glucose deprivation has been shown to enhance phosphorylation of eIF2α, leading to increases in levels of BACE1 and consequently Aβ accumulation^14^. We found that the peripheral surgery increased the levels of P-eIF2α, BACE1, and Aβ in the hippocampus of aged WT mice (Figure 4 and 6). These findings suggested that the peripheral surgery may also induce cellular stress, ultimately leading to Aβ accumulation.

Finally, γ-secretase inhibitor compound E (Figure 7), which reduces Aβ generation^15^, could mitigate the peripheral surgery-induced Aβ accumulation and cognitive impairment in the 18 month-old WT mice. These findings demonstrated the potential cause-effect relationship of the peripheral surgery-induced Aβ accumulation and cognitive impairment. Furthermore, these findings suggested the potential application of future anti-Aβ treatment in preventing and treating POCD, pending further studies. There was a moderate increase in the Aβ40 levels in the mouse hippocampus following the treatment of compound E. The increase could be non-specific, however, the exact reason of this increase remained unknown. Nevertheless, the data suggested that compound E might not reduce brain Aβ levels in the absence of surgery insult, but could mitigate the surgery-induced elevation of brain Aβ levels.

POCD may result from the surgery-induced neuroinflammation, including elevation of brain levels of pro-inflammatory cytokine and microglia activation [7,8, reviewed in^10^]. Specifically, neuroinflammation may cause cognitive dysfunction through synaptic dysfunction, inhibition of neurogenesis, neuronal death, microglial priming and others, contributing to POCD [reviewed in^10^]. Aβ and neuroinflammation have been reported to potentiate each other's neurotoxicity [31,32; reviewed in^33^]. We therefore have proposed a multifactorial model of POCD pathogenesis that neuroinflammation from surgery plus Aβ accumulation from aging or AD gene mutation are needed to cause POCD. Future studies are needed to test this hypothesis.

Stress^34^ and pain^35^ may cause cognitive dysfunction [reviewed in^36^]. The mice in the current studies underwent the peripheral surgery with only local anesthesia, and the mice were restrained with paper tape for the procedure. Thus, we cannot completely rule out the effects of stress and pain on the peripheral surgery-induced neurotoxicity and neurobehavioral deficits in the mice. Indeed, there was a slight increase in the levels of blood glucose (but not epinephrine) and the pain scale in surgery mice than those in the control mice (Table 1), which indicated that the peripheral surgery may also induce minimal levels of stress response and pain. However, there have been no other alternative ways to determine the effects of peripheral surgery without the influence of the general anesthesia on the cognitive impairment and the underlying mechanisms. These findings would hopefully promote more studies of POCD, including whether the peripheral surgery-associated pain and stress also contribute to the development of POCD.

In conclusion, we reported that the peripheral surgery in mice abdomen, in the absence of general anesthetics (under local anesthesia), induced brain Aβ accumulation and cognitive impairment in aged WT mice and AD Tg mice, but not adult WT mice. P-eIF2α, BACE1, and Aβ accumulation might all be involved as, at least partially, the underlying mechanisms. Finally, γ-secretase inhibitor (compound E) ameliorated the peripheral surgery-induced adverse effects. Taken together, these findings carry insightful implications for the surgical care of elderly patients and suggest that the peripheral surgery combined with age or AD gene mutation-associated Aβ accumulation may contribute to POCD. Pending further studies, it may be useful to consider future anti-Aβ therapies to reduce the risk of POCD in elderly patients and, particularly, those suffering from AD.

## Methods

### Mice surgery and treatment

All experiments were performed in accordance with the National Institutes of Health guidelines and regulations. The animal protocol was approved by the Massachusetts General Hospital (Boston, Massachusetts) Standing Committee on the Use of Animals in Research and Teaching. Efforts were made to minimize the number of animals used. Since it is technically difficult to perform an epidural or spinal anesthesia in mice, we have established an animal model of peripheral surgery in the abdomen under local anesthesia in mice. WT C57BL/6J mice (9 month-old, The Jackson Laboratory, Bar Harbor, ME; and 18 month-old, National Institute of Aging, Bethesda, MD), and AD Tg mice [B6.Cg-Tg(APPswe, PSEN1dE9) 85Dbo/J, 9 month-old, The Jackson Laboratory] were used in the studies. The mice were randomly assigned to a surgery or control group by weight. The mice were gently restrained to a heating pad (37 C°) using paper tape. A local anesthetic bupivacine (0.5% and 0.1 ml) was injected into the skin and subcutaneous tissue of the abdominal area. A 2.5 cm incision was made in the middle of the abdomen to open and then close the abdominal cavity in the mouse. The procedure lasted about five minutes. We did not use sedative medicine in an effort to reveal the effects of surgery alone and to minimize all other variables. EMLA cream (2.5% lidocaine and 2.5% prilocaine) was used every 8 hours for the first and second post-operative days to treat the surgery-associated pain. We did not use antibiotics because the procedure was aseptic. The non-surgery (control) mice underwent the same procedure, only without the incision. In the intervention studies, each mouse received compound E (the inhibitor of γ-secretase, which can reduce Aβ generation) (3 mg/kg, IP, Enzo Life Sciences Inc., Farmingdale, NY, Cat. Number: ALX-270-415) or saline daily for 7 days post-surgery^20^.

### Measurement of physiological changes in mice receiving peripheral surgery

A mouse-tail blood pressure cuff (Kent scientific cooperation, Torrington, CT) was used to measure blood pressure. Blood gas and blood glucose levels were determined by a blood gas machine (Trupoint, ITC, Edison, NJ). Blood epinephrine levels were determined by enzyme-linked immunosorbent assay (ELISA) kit (American research products, Inc., Belmont, MA). Locomotor activity was counted by a recorded video of the mice. Pain threshold was determined by Von Frey fiber (North Coast Medical, Inc., Gilroy, CA) as described in a previous study^37^. The Von Frey fiber was applied to the abdominal wound to assess the pain threshold.

### Fear conditioning system (FCS)

The FCS studies were performed as described in our previous studies^6^ with modification. Specifically, the pairing in the FCS (Stoelting Co., Wood Dale, IL) was performed 24 hours post-surgery, mimicking the condition that patients may have difficulty to learn new things after surgery. For pairing, each mouse was allowed to explore the FCT chamber for 180 seconds before presentation of a 2-Hz pulsating tone (80 dB, 3,600 Hz) that persisted for 60 seconds. The tone was immediately followed by a mild foot shock (0.8 mA for 0.5 second). The pairing was performed twice with two minutes in between. The first context test of FCT was performed at 30 minutes after the end of the pairing. Each mouse was allowed to stay in the same chamber for a total of 390 seconds. Cognitive function (e.g., learning and memory) in the context test was assessed by measuring the amount of time the mouse demonstrated “freezing behavior” (freezing time), which is defined as a completely immobile posture except for respiratory efforts during the second 180 seconds. The first tone test was performed at 90 minutes after the end of the pairing. Each mouse was allowed to stay in a different chamber for a total of 390 seconds. The same tone was presented for the second 180 seconds without the foot shock. Cognitive function in the tone test was also assessed by measuring the freezing time. The same cohorts of 9 and 18 month-old mice were tested repeatedly in the FCT at 24 and 72 hours, 7, 30 and 60 days post-surgery without additional pairing. This design was consistent with the observation that patients may have specific cognitive impairments for extended periods of time post-surgery. To control for sequential testing of the same mice, we observed surgery-induced cognitive impairment at 7 days post-surgery, independent of repeating the FCT test (Figure 7). We used a double pairing method in order to illustrate that the peripheral surgery could impair cognition over a long period of time (e.g., 60 days) post-surgery. However, this method caused 9 month-old mice to exhibit relative hyperactivity in the context test of the FCS, which led to a shorter freezing time, a caveat of the method.

### Morris water maze (MWM)

Both 9 and 18 month-old mice (different cohort from the mice in FCS studies) from the peripheral surgery and control group were tested in the MWM (Stoelting Co.) starting on the next day after the peripheral surgery as previously described^22^. The mice were placed in MWM one day after the surgery. We dried the wound of the mice immediately after each trail of MWM test, and no sign of infection in the mice was identified in the studies. We did not include a training period for both FCS and MWM studies because we wanted to specifically determine whether surgery could impair the cognitive function of mice in learning new things. The similar approach was employed in other studies^5^,^22^.

### Brain tissue lysis and protein quantification

The brain tissues (hippocampus) of the mice were harvested at 12 hours after the surgery. The harvested brain tissues were homogenized on ice using immunoprecipitation buffer (10 mM Tris-HCl, pH 7.4, 150 mM NaCl, 2 mM EDTA, 0.5% Nonidet P-40) plus protease inhibitors (1 μg/ml aprotinin, 1 μg/ml leupeptin, 1 μg/ml pepstatin A). The lysates were collected, centrifuged at 12,000 rpm for 15 minutes, and quantified for total proteins by bicinchoninic acid (BCA) protein assay kit (Pierce, Iselin, NJ). The brain tissues were then subjected to Western blot analysis as described by Xie et al.^38^.

### Western blot analysis

6E10 antibody (1:200 dilution; Covance, Princeton, NJ, Cat. Number: SIG-39320) was used to recognize Aβ (4 kDa). Beta-site amyloid precursor protein cleaving enzyme 1(BACE1) antibody (1:1,000 dilution; Abcam, Cambridge, MA, Cat. Number: ab2077) was used to recognize BACE1 (65 kDa). Phospho-eukaryotic translation initiation factor (eIF) 2α antibody (Ser51, 119A11) (1:1,000 dilution, Cell Signaling, Cat. Number: 3597) was used to recognize phosphorylated (eIF) 2α, (P-eIF2α) (38 kDa). Antibody anti-β-Actin (1:10,000, Sigma, St. Louis, MO) was used to detect β-Actin (42 kDa) levels. Western blot quantification was performed as described by Xie et al.^38^. 100% of protein level changes refer to control levels for the purpose of comparison to experimental conditions.

### Enzyme-linked immunosorbent assay (ELISA) Aβ measurement

The levels of Aβ40 and Aβ42 were measured by using ELISA (Invitrogen, San Francisco, CA) as described in our previous studies^39^. The mouse Aβ40 and Aβ42 immunoassay Kits (Invitrogen, Catalog number: KMB3481 and KMB 3441) were used to determine Aβ40 and Aβ42 levels, respectively, in the hippocampus of the 9 and 18 month-old WT mice.

### Immunoblot detection of Aβ

Immunoblot detection of Aβ in hippocampus was measured as described in previous studies^38^,^40^,^41^. Specifically, brain samples were homogenized (150 mM NaCl with protease inhibitor cocktail in 50 mM Tris, pH of 8.0) and centrifuged (65,000 rpm × 45 minutes), and the supernatant was removed. The pellet was then resuspended by sonication in homogenization buffer containing 1% SDS. Following pelleting of insoluble material (18,000 rpm × 15 minutes), the SDS-extract was electrophoresed on SDS-PAGE (4–12% Bis-Tris polyacrylamide gel from Invitrogen), blotted to PVDF membrane and probed with a 1:200 dilution of 6E10 antibody (Covance).

### Statistics

The nature of the hypothesis testing was two-tailed. Data were expressed as mean ± Standard Error of the Mean (SEM). The data for platform crossing times were not normally distributed, thus were expressed as median and interquartile range (IQR, 25% to 75%). The number of samples varied from 6 (biochemistry studies) to 10 (behavioral studies). Two-tailed t-test and one-way ANOVA were used to compare the differences between groups. Interaction between time and group factors in a two-way ANOVA with repeated measurements was used to analyze the interaction of age and time between mice in the control group and peripheral surgery group in the MWM. Two-way ANOVA was also used to determine the interaction of surgery and age or AD gene mutation effects in the studies. There were no missing data for the variables of MWM (escape latency and platform crossing times) during the data analysis. Finally, the Mann-Whitney test was used to determine the difference in platform crossing times between the peripheral surgery and control conditions. P values less than 0.05 (*, # and ^∧^) and 0.01 (**, ## and ^∧∧^) were considered statistically significant. Prism 6 software (La Jolla, CA, USA) and SAS (SAS Institute Inc, Cary, NC) software (version 9.2) were used for all statistical analyses.

## Author Contributions

Z.X., D.C., G.C., E.R. and R.T. conceived and designed the project. Z.Xu., Y.D., H.W., Y.Z. and Z.X. performed all the experiments and prepared the figures. Z.X. wrote the manuscript. All authors reviewed the manuscript.

## Supplementary Material

Supplementary Information Supplementary Figures

## Figures and Tables

**Table 1:** The physiological changes in the mouse model of surgery

	Control Mice (wild-type) (9 month-old)	Surgery Mice (wild-type) (9 month-old)
**Mean arterial pressure (MAP) (mmHg)**	117 ± 1.74	114 ± 1.79
**pH**	7.41 ± 0.03	7.36 ± 0.04
**PaO2 (mmHg)**	182 ± 4.44	172 ± 4.90
**PaCO2 (mmHg)**	41.2 ± 1.08	39.8 ± 0.45
**Blood glucose levels (mg/dl)**	110 ± 1.28	132 ± 6.88
**Blood epinephrine levels (ng/ml)**	91.2 ± 10.38	94.7 ± 5.12
**Locomotor activity (1 day) (move/min)**	59.6 ± 2.39	57.2 ± 2.24
**Pain threshold (1 day) (gram)**	9.7 ± 2.06	8.0 ± 2.47

C57BL/6J mice received the peripheral surgery as described in the methods section. As compared to the control mice, the peripheral surgery mice did not show significant changes in behavior (e.g., eating and drinking), intraoperative blood pressure, blood gas, post-operative locomotor activity, blood glucose levels, blood epinephrine levels, and the pain threshold. Values are expressed as mean ± SEM. N = 6–10.

## References

[b1] Liu L. L. & Leung J. M. Predicting adverse postoperative outcomes in patients aged 80 years or older. J Am Geriatr Soc 48, 405–412 (2000). 10798467 10.1111/j.1532-5415.2000.tb04698.x

[b2] Monk T. G. *et al.* Predictors of cognitive dysfunction after major noncardiac surgery. Anesthesiology 108, 18–30 (2008). 18156878 10.1097/01.anes.0000296071.19434.1e

[b3] Deiner S. & Silverstein J. H. Postoperative delirium and cognitive dysfunction. Br J Anaesth 103 Suppl 1, i41–46 (2009). 20007989 10.1093/bja/aep291 PMC2791855

[b4] Steinmetz J., Christensen K. B., Lund T., Lohse N. & Rasmussen L. S. Long-term consequences of postoperative cognitive dysfunction. Anesthesiology 110, 548–555 (2009). 19225398 10.1097/ALN.0b013e318195b569

[b5] Saab B. J. *et al.* Short-term memory impairment after isoflurane in mice is prevented by the alpha5 gamma-aminobutyric acid type A receptor inverse agonist L-655,708. Anesthesiology 113, 1061–1071 (2010). 20966663 10.1097/ALN.0b013e3181f56228

[b6] Zhang Y. *et al.* Anesthetics isoflurane and desflurane differently affect mitochondrial function, learning, and memory. Ann Neurol 71, 687–698 (2012). 22368036 10.1002/ana.23536 PMC3942786

[b7] Terrando N. *et al.* Tumor necrosis factor-alpha triggers a cytokine cascade yielding postoperative cognitive decline. Proc Natl Acad Sci U S A 107, 20518–20522 (2010). 21041647 10.1073/pnas.1014557107 PMC2996666

[b8] Wan Y. *et al.* Cognitive decline following major surgery is associated with gliosis, beta-amyloid accumulation, and tau phosphorylation in old mice. Crit Care Med 38, 2190–2198 (2010). 20711073 10.1097/CCM.0b013e3181f17bcb

[b9] Newman S., Stygall J., Hirani S., Shaefi S. & Maze M. Postoperative cognitive dysfunction after noncardiac surgery: a systematic review. Anesthesiology 106, 572–590 (2007). 17325517 10.1097/00000542-200703000-00023

[b10] Lyman M., Lloyd D. G., Ji X., Vizcaychipi M. P. & Ma D. Neuroinflammation: The role and consequences. Neurosci Res (In Press). 10.1016/j.neures.2013.10.004 24144733

[b11] Querfurth H. W. & LaFerla F. M. Alzheimer's disease. N Engl J Med 362, 329–344 (2010). 20107219 10.1056/NEJMra0909142

[b12] Garcia-Alloza M. *et al.* Characterization of amyloid deposition in the APPswe/PS1dE9 mouse model of Alzheimer disease. Neurobiol Dis 24, 516–524 (2006). 17029828 10.1016/j.nbd.2006.08.017

[b13] Xiong H. *et al.* Biochemical and behavioral characterization of the double transgenic mouse model (APPswe/PS1dE9) of Alzheimer's disease. Neurosci Bull 27, 221–232 (2011). 21788993 10.1007/s12264-011-1015-7 PMC5560305

[b14] O'Connor T. *et al.* Phosphorylation of the translation initiation factor eIF2alpha increases BACE1 levels and promotes amyloidogenesis. Neuron 60, 988–1009 (2008). 19109907 10.1016/j.neuron.2008.10.047 PMC2667382

[b15] Grimwood S. *et al.* Determination of guinea-pig cortical gamma-secretase activity ex vivo following the systemic administration of a gamma-secretase inhibitor. Neuropharmacology 48, 1002–1011 (2005). 15857627 10.1016/j.neuropharm.2005.01.016

[b16] Morris R. Developments of a water-maze procedure for studying spatial learning in the rat. J Neurosci Methods 11, 47–60 (1984). 6471907 10.1016/0165-0270(84)90007-4

[b17] Teeling J. L. & Perry V. H. Systemic infection and inflammation in acute CNS injury and chronic neurodegeneration: underlying mechanisms. Neuroscience 158, 1062–1073 (2009). 18706982 10.1016/j.neuroscience.2008.07.031

[b18] Gaykema R. P. *et al.* Bacterial endotoxin induces fos immunoreactivity in primary afferent neurons of the vagus nerve. Neuroimmunomodulation 5, 234–240 (1998). 9730691 10.1159/000026343

[b19] Fukumoto H. *et al.* Beta-secretase activity increases with aging in human, monkey, and mouse brain. Am J Pathol 164, 719–725 (2004). 14742275 10.1016/s0002-9440(10)63159-8 PMC1602259

[b20] Hong S. *et al.* Dynamic analysis of amyloid beta-protein in behaving mice reveals opposing changes in ISF versus parenchymal Abeta during age-related plaque formation. J Neurosci 31, 15861–15869 (2011). 22049429 10.1523/JNEUROSCI.3272-11.2011 PMC3227224

[b21] Culley D. J., Baxter M. G., Yukhananov R. & Crosby G. Long-term impairment of acquisition of a spatial memory task following isoflurane-nitrous oxide anesthesia in rats. Anesthesiology 100, 309–314 (2004). 14739805 10.1097/00000542-200402000-00020

[b22] Bianchi S. L. *et al.* Brain and behavior changes in 12-month-old Tg2576 and nontransgenic mice exposed to anesthetics. Neurobiol Aging 29, 1002–1010 (2008). 17346857 10.1016/j.neurobiolaging.2007.02.009 PMC4899817

[b23] Tang J., Eckenhoff M. F. & Eckenhoff R. G. Anesthesia and the old brain. Anesth Analg 110, 421–426 (2010). 19820235 10.1213/ANE.0b013e3181b80939 PMC13498403

[b24] Wan Y. *et al.* Postoperative impairment of cognitive function in rats: a possible role for cytokine-mediated inflammation in the hippocampus. Anesthesiology 106, 436–443 (2007). 17325501 10.1097/00000542-200703000-00007

[b25] Terrando N. *et al.* The impact of IL-1 modulation on the development of lipopolysaccharide-induced cognitive dysfunction. Crit Care 14, R88 (2010). 20470406 10.1186/cc9019 PMC2911722

[b26] Cibelli M. *et al.* Role of interleukin-1beta in postoperative cognitive dysfunction. Ann Neurol 68, 360–368 (2010). 20818791 10.1002/ana.22082 PMC4836445

[b27] Rasmussen L. S. *et al.* Does anaesthesia cause postoperative cognitive dysfunction? A randomised study of regional versus general anaesthesia in 438 elderly patients. Acta Anaesthesiol Scand 47, 260–266 (2003). 12648190 10.1034/j.1399-6576.2003.00057.x

[b28] Williams-Russo P., Sharrock N. E., Mattis S., Szatrowski T. P. & Charlson M. E. Cognitive effects after epidural vs general anesthesia in older adults. A randomized trial. JAMA 274, 44–50 (1995). 7791257

[b29] Mason S. E., Noel-Storr A. & Ritchie C. W. The impact of general and regional anesthesia on the incidence of post-operative cognitive dysfunction and post-operative delirium: a systematic review with meta-analysis. J Alzheimers Dis 22 Suppl 3, 67–79 (2010). 20858956 10.3233/JAD-2010-101086

[b30] Moller J. T. *et al.* Long-term postoperative cognitive dysfunction in the elderly ISPOCD1 study. ISPOCD investigators. International Study of Post-Operative Cognitive Dysfunction. Lancet 351, 857–861 (1998). 9525362 10.1016/s0140-6736(97)07382-0

[b31] Yamamoto M. *et al.* Interferon-gamma and tumor necrosis factor-alpha regulate amyloid-beta plaque deposition and beta-secretase expression in Swedish mutant APP transgenic mice. Am J Pathol 170, 680–692 (2007). 17255335 10.2353/ajpath.2007.060378 PMC1851864

[b32] Liao Y. F., Wang B. J., Cheng H. T., Kuo L. H. & Wolfe M. S. Tumor necrosis factor-alpha, interleukin-1beta, and interferon-gamma stimulate gamma-secretase-mediated cleavage of amyloid precursor protein through a JNK-dependent MAPK pathway. J Biol Chem 279, 49523–49532 (2004). 15347683 10.1074/jbc.M402034200

[b33] Selkoe D. J. Alzheimer's disease: genes, proteins, and therapy. Physiol Rev 81, 741–766 (2001). 11274343 10.1152/physrev.2001.81.2.741

[b34] Marcos B., Aisa B. & Ramirez M. J. Functional interaction between 5-HT(6) receptors and hypothalamic-pituitary-adrenal axis: cognitive implications. Neuropharmacology 54, 708–714 (2008). 18206183 10.1016/j.neuropharm.2007.11.019

[b35] Hu Y., Yang J., Wang Y. & Li W. Amitriptyline rather than lornoxicam ameliorates neuropathic pain-induced deficits in abilities of spatial learning and memory. Eur J Anaesthesiol 27, 162–168 (2010). 19915478 10.1097/EJA.0b013e328331a3d5

[b36] Shansky R. M. & Lipps J. Stress-induced cognitive dysfunction: hormone-neurotransmitter interactions in the prefrontal cortex. Frontiers in human neuroscience 7, 123 (2013). 23576971 10.3389/fnhum.2013.00123 PMC3617365

[b37] Chaplan S. R., Bach F. W., Pogrel J. W., Chung J. M. & Yaksh T. L. Quantitative assessment of tactile allodynia in the rat paw. J Neurosci Methods 53, 55–63 (1994). 7990513 10.1016/0165-0270(94)90144-9

[b38] Xie Z. *et al.* The common inhalation anesthetic isoflurane induces caspase activation and increases amyloid beta-protein level in vivo. Ann Neurol 64, 618–627 (2008). 19006075 10.1002/ana.21548 PMC2612087

[b39] Lu Y. *et al.* Anesthetic sevoflurane causes neurotoxicity differently in neonatal naive and Alzheimer disease transgenic mice. Anesthesiology 112, 1404–1416 (2010). 20460993 10.1097/ALN.0b013e3181d94de1 PMC2877754

[b40] Nagano S. *et al.* Peroxidase activity of cyclooxygenase-2 (COX-2) cross-links beta-amyloid (Abeta) and generates Abeta-COX-2 hetero-oligomers that are increased in Alzheimer's disease. J Biol Chem 279, 14673–14678 (2004). 14724276 10.1074/jbc.M313003200

[b41] Dong Y. *et al.* The common inhalational anesthetic sevoflurane induces apoptosis and increases beta-amyloid protein levels. Arch Neurol 66, 620–631 (2009). 19433662 10.1001/archneurol.2009.48 PMC2748878

