# Supplementary material for: Age-dependent postoperative cognitive impairment and Alzheimer-related neuropathology in mice
Source: Sci Rep. 2014 Jan 20;4:3766. doi: 10.1038/srep03766 (PMC3895908; doi:10.1038/srep03766)
Supplement: Supplementary Information — Supplementary Figures [file srep03766-s1.pdf]

Supplementary Materials for

Age-dependent postoperative cognitive impairment and Alzheimer-related neuropathology in mice

Zhipeng Xu [1,#], Yuanlin Dong [1,#], Hui Wang [1,2], Deborah J. Culley [3], Edward R. Marcantonio, [4], Gregory Crosby [3], Rudolph E. Tanzi [5], Yiying Zhang [1] and Zhongcong Xie [1*]

*To whom correspondence should be addressed. E-mail: zxie@mgh.harvard.edu

This file includes:
Supplementary Figure1 and Figure 2

Supplementary Figures

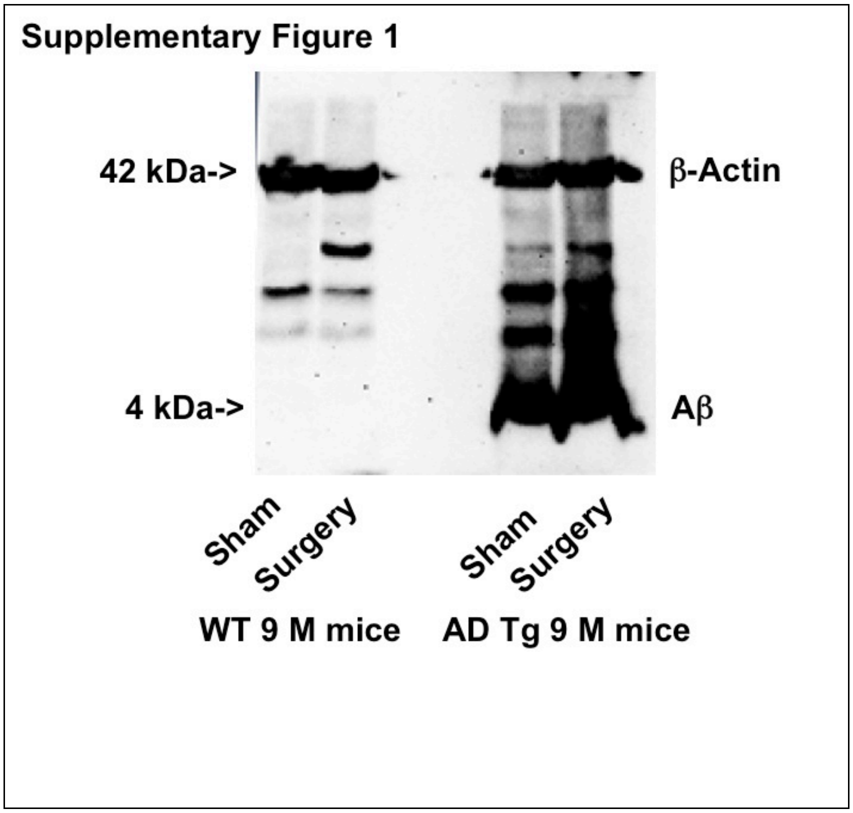

Supplementary Figure 1. The baseline Aβ levels in the hippocampus of the 9 month-old AD Tg mice are higher than those in 9 month-old WT mice, and the peripheral surgery increases the hippocampus Aβ levels in the 9 month-old AD Tg mice but not in the 9 month-old WT mice.

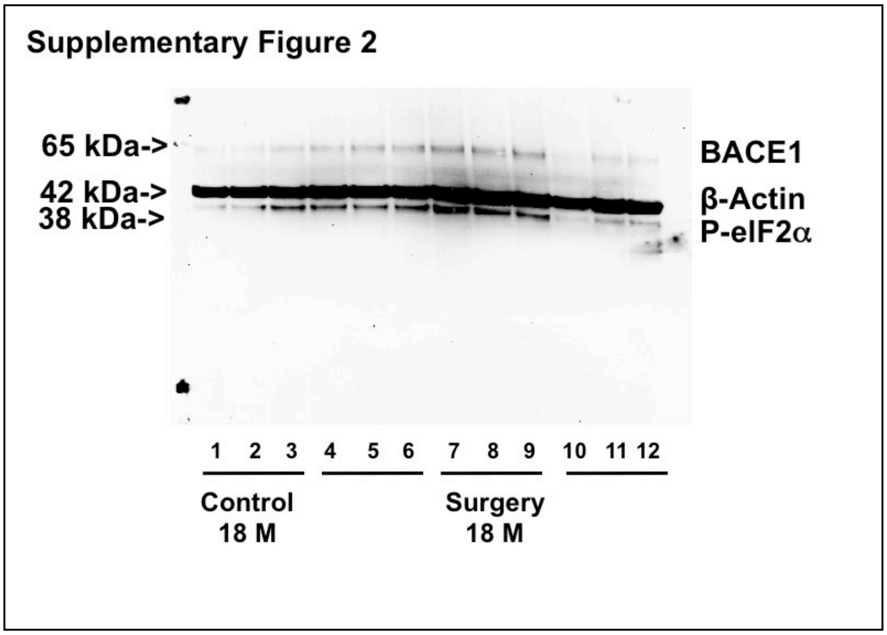

Supplementary Figure 2. Peripheral surgery (bands 7 to 9) increases BACE1 and P-eIF2α levels in the hippocampus of 18 month-old mice at 12 hours post-surgery as compared to the control condition (bands 1 to 3).
